# Effect of positive end-expiratory pressure on porcine right ventricle function assessed by speckle tracking echocardiography

**DOI:** 10.1186/s12871-015-0028-6

**Published:** 2015-04-11

**Authors:** Sam R Orde, Atta Behfar, Paul G Stalboerger, Sergio Barros-Gomes, Garvan C Kane, Jae K Oh

**Affiliations:** 1 Division of Cardiovascular Diseases, Mayo Clinic, 200 First St SW, Rochester, MN 55905 USA; 2 Intensive Care Unit, Nepean Hospital, Kingswood, Sydney, 2749 NSW Australia

**Keywords:** Speckle tracking echocardiography, Right ventricle, Right ventricle strain, PEEP, Mechanical ventilation

## Abstract

**Background:**

Right ventricle (RV) dysfunction and hypotension can be induced by high levels of positive end-expiratory pressure (PEEP). We sought to determine in an animal model if a novel ultrasound analysis technique: speckle tracking echocardiography (STE), could determine deterioration in RV function induced by PEEP and to compare this to a conventional method of RV analysis: fractional area change (FAC). STE is a sensitive, angle-independent method for describing cardiac deformation (‘strain’) and is particularly useful in analyzing RV function as has been shown in pulmonary hypertension cohorts.

**Methods:**

Ten pigs, 40-90 kg, anaesthetized, fully mechanically ventilated at 6 ml/kg were subject to step-wise escalating levels of PEEP at two-minute intervals (0, 5, 10, 15, 20, 25 and 30cmH_2_0). Intracardiac echocardiography was used to image the RV as transthoracic and transesophageal echocardiography did not give sufficient image quality or flexibility. Off-line STE analysis was performed using Syngo Velocity Vector Imaging (Seimens Medical Solutions Inc., USA). STE systolic parameters are RV free wall strain (RVfwS) and strain rate (RVfwSR) and the diastolic parameter RV free wall strain rate early relaxation (RVfwSRe)

**Results:**

With escalating levels of PEEP there was a clear trend of reduction in STE parameters (RVfwS, RVfwSR, RVfwSRe) and FAC. Significant hypotension (fall in mean arterial blood pressure of 20 mmHg) occurred at approximately PEEP 15 cmH_2_O. Comparing RVfwS, RVfwSR and RVfwSRe values at different PEEP levels showed a significant difference at PEEP 0 cmH_2_O vs PEEP 10 cmH_2_O and above. FAC only showed a significant difference at PEEP 0 cmH_2_O vs PEEP 20 cmH_2_O and above. 30% of pigs displayed dyssychronous RV free wall contraction at the highest PEEP level reached.

**Conclusions:**

STE is a sensitive method for determining RV dysfunction induced by PEEP and deteriorated ahead of a conventional assessment method: FAC. RVfwS decreased to greater extent compared to baseline than FAC, earlier in the PEEP escalation process and showed a significant decrease before there was a clinical relevant decrease in mean arterial blood pressure. Studies in ICU patients using transthoracic echocardiography are warranted to further investigate the most sensitive echocardiography method for detecting RV dysfunction induced by mechanical ventilation.

## Background

Right ventricle (RV) failure in the critically ill is an independent risk factor for mortality in patients with acute lung injury and acute respiratory distress syndrome (ARDS) [1,2]. It can be challenging to treat and requires accurate and early recognition in order to tailor treatment [3,4]. Echocardiography has a crucial role in the diagnosis of RV failure in the ICU [5]. Interpretation can be difficult however, due to the crescentric shape, retrosternal position and the poor correlation between conventional assessment methods, such as fractional area change (FAC) and intrinsic RV contractile dysfunction [6] as well as translational errors with methods such as tricuspid annular plane systolic excursion and tissue Doppler imaging. Speckle tracking echocardiography (STE) has emerged as a relatively novel, angle-independent technique for analyzing the grey-scale ultrasound (B mode) images of the heart [7] and can elucidate cardiac dysfunction not seen with conventional echocardiography techniques [8,9]. STE is particularly useful for assessing RV systolic function: RV free wall strain (RVfwS) and RV free wall strain rate (RVfwSR) which are suggested to be more robust measures of RV contractility than conventional echocardiography methods in diseases such as pulmonary hypertension [10-13].

Positive end expiratory pressure (PEEP) is an integral component of mechanical ventilation in critically ill patients suffering from acute lung injury and ARDS, yet can have negative consequences on cardiac haemodynamics [14]. ‘Open-lung ventilation’ aims to decrease the cyclic opening and closing of small distal airways and atelectatic alveoli which can lead to ventilator-induced lung injury [15,16] through the use of elevated PEEP levels. Cardiac function can be affected by high PEEP levels in several ways including: biventricular reduced venous return and increased right ventricle (RV) afterload, which is poorly tolerated [17] resulting in RV dysfunction, cor pulmonale and acute hypotension [3].

The aim of this study was to perform a step-wise PEEP escalation maneuver in anesthetized, fully mechanically ventilated pigs and to assess their RV function with STE and a conventional echocardiography measure of RV function analysis: FAC. We sought to 1) Determine if STE could describe changes in RV function induced by escalating levels of PEEP; 2) To compare RVfwS to a conventional measure of RV function assessment: FAC; and 3) Determine if RVfwS or FAC deterioration occurred prior to PEEP induced hypotension (defined as a fall in mean arterial blood pressure [MAP] of 20 mmHg).

## Methods

All animal experiments and protocols were approved and carried out according to the guidelines of the Animal Care and Use Committee of the Mayo Clinic (Rochester, MN, USA). In 10 Yorkshire female swine weighing median 45 kg (41.5 to 60.5 interquartile range [IQR]), after overnight fasting, anesthesia was induced with Telazol (5 mg/kg intramuscularly) and Xylazine (2.0 mg/kg intramuscularly). The animals were intubated with a 7 mm internal diameter endotracheal tube, mechanically ventilated by a Datex-Ohmeda 7100 ventilator (GE Healthcare) with volume control mode at tidal volumes of 6 ml/kg, fraction of inspired oxygen (FiO2) started at 0.4 aiming for saturations greater than 92%, inspiratory/expiratory ratio of 1:2, end-inspiratory pause of 10%, respiratory rate of 16 breaths per minute and initial PEEP of 0 cmH_2_O. Anesthesia was maintained with inhaled isoflurane 1.0-3.0%. The pigs were placed in a supine position during the entire experiment.

**Figure 1:**
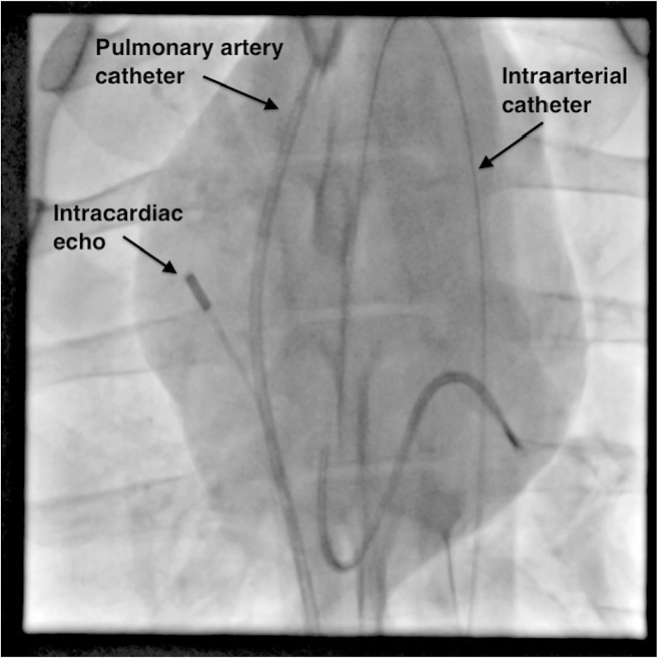
**Fluroscopy images of intracardiac echocardiography (ICE), pulmonary artery and arterial catheters used during the study.**

Percutaneous access was achieved through the femoral artery and both femoral veins for monitoring of arterial pressures (9 F sheath), pulmonary artery pressures (831HF75P, Swan-Ganz 7.5 French, Edwards Life-sciences, Irvine, CA) and for the intracardiac echocardiography (ICE) catheter respectively. Correct positioning of the pulmonary artery catheter in the pulmonary artery was confirmed with fluoroscopy (see Figure 1), waveform analysis and by inflation of the catheter balloon. All intravascular catheters were zeroed to the atmosphere. The mid-point of the anterior and posterior chest was considered the reference point. Electrocardiogram and intravascular pressures were monitored continuously.

**Figure 2:**
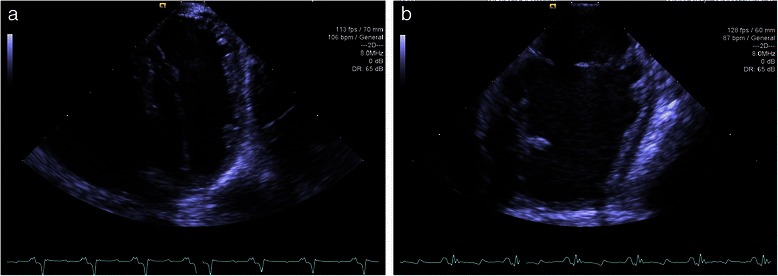
**Representative intracardiac echocardiogram (ICE) images of the right ventricle (RV) at end-expiration and end-diastole. (a)** PEEP 0 and **(b)** PEEP 30. Imaging optimized to assess the RV free wall, including the tricuspid annulus throughout the cardiac cycle, maximizing for frame rate to allow for accurate speckle tracking assessment.

Due to the mediastinal anatomy of the swine, transthoracic and transoesophageal echocardiography did not provide sufficient image quality or flexibility to sufficiently image the RV free wall. We therefore had to use ICE, performed with an 8 French AcuNav ultrasound catheter connected to an Acuson SC2000 ultrasound machine (Siemens Medical USA, Malvern, Pennsylvania) inserted into the right atrium. Imaging was performed by A.B. (who is appropriately trained in this method) and was optimized for maximal frame rate to enable accurate speckle tracking and focused on the RV free wall in the long axis ensuring the tricuspid annulus was visible throughout the cardiac cycle (see Figure 2). The mean (±SD) frame rate was 113 (±13) frames per second.

**Figure 3:**
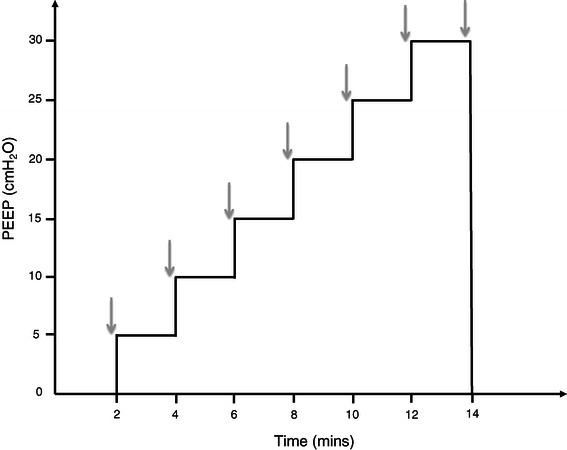
**Graphical representation of the step-wise escalating PEEP protocol.** Indicates timing of recordings made at end-expiration: physiological data and intracardiac echocardigraphy (ICE) images for post processing analysis.

Once baseline stability was achieved, PEEP was increased in a stepwise manner every 2 minutes from PEEP 0 cmH_2_O to PEEP 30cmH_2_O in 5cmH_2_O increments keeping the tidal volumes constant (see Figure 3). Before each increase in PEEP, recordings were made at end-expiration: intracardiac echocardiography clips of 3 seconds, haemodynamic parameters and saturations. Significant hypotension was considered a decrease in MAP of 20 mmHg. The stepwise PEEP maneuver was ceased at PEEP 30 cmH_2_O and PEEP returned to 0 cmH_2_O. The maneuver was ceased earlier if MAP fell below 25 mmHg, heart rate fell below 40 beats per minute or if oxygen saturation fell below 60% and was unresponsive to FiO2 of 1.0.

**Figure 4:**
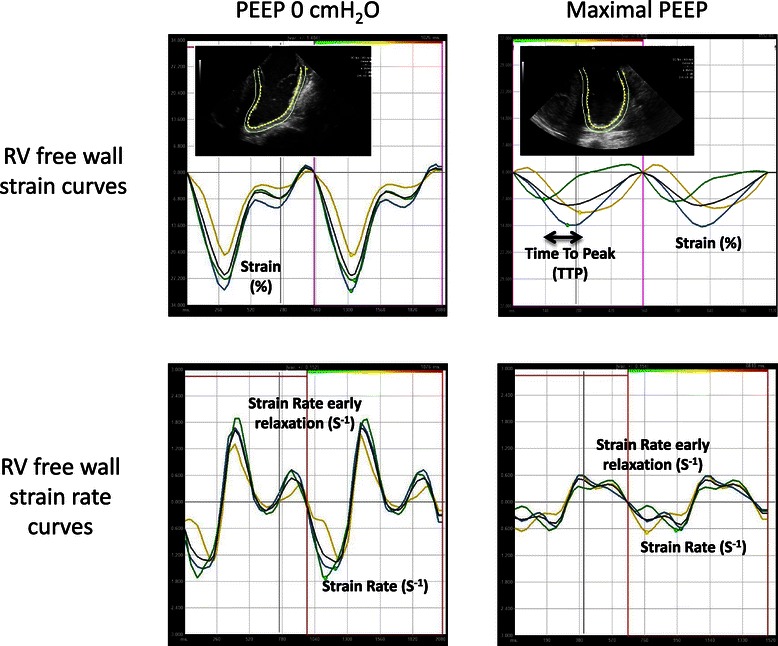
**Examples of strain and strain rate curves of the right ventricle free wall segments.** Each right ventricle free wall segment (basal, mid and apex) is represented with a curve (black curve = mean value). Examples of strain and strain rate curves at baseline positive end-expiratory pressure (PEEP 0 cmH_2_O) and at maximal PEEP represented. At higher levels of PEEP both RV systolic function parameters (RV free wall Strain [RVfwS] and RV free wall Strain Rate [RVfwSR]) as well as RV diastolic function parameters (RV free wall Strain Rate early relaxation [RVfwSRe]) showed deterioration. Time To Peak strain (TTP) indicates delay in time from first segment to reach maximal strain value to last segment to reach maximal strain value. TTP can be used to determine dyssynchrony of segmental contraction.

Two-dimensional ICE images were transferred to a Syngo Velocity Vector Imaging workstation (Siemens Medical USA, Malvern, Pennsylvania). A single best cardiac cycle was chosen to determine FAC by manually tracing the RV endocardium at end-diastole and end-systole: FAC = ([end diastolic area – end systolic area]/end-diastolic area) x 100. One to three beat cardiac cycles were chosen for STE analysis. The endocardium was traced manually at end-systole from the medial to lateral annulus with approximately 7–15 points. RV function values are an average of the three free wall segments. Systolic function parameters are measures of deformation or strain: RVfwS and RVfwSR (change in strain/time) and these are negative values: the more negative the value the better the function. Diastolic function is determined by a positive value: RV free wall strain rate early relaxation (RVfwSRe): the speed that deformation returns to the end-diastolic value. Strain and strain rate curves were chosen based on appropriate tracking as well as assessing displacement, velocity, strain and strain rate curves for appropriate motion, smoothness and segment correlation. The same cardiac cycle was chosen for strain and strain rate values. See Figure 4 for examples of strain and strain rate curves at PEEP 0 cmH_2_0 and final PEEP value.

To assess for synchrony of RV free wall segment contraction we used a method proposed by Yu et al. [18] to assess for dyssynchrony of the left ventricle. The Time To Peak (TTP) strain value is determined by comparing the time taken from the onset of the QRS to peak strain value for each segment. TTP delay is the time difference between the segments with the smallest TTP compared to segment with the largest (see Figure 4: Maximal PEEP strain curve). The mean of the TTP delay at PEEP 0 cmH_2_O was used as the reference value and 2 standard deviations was added to this to obtain the 95^th^ percentile value. We then assessed the TTP delay at the highest PEEP level that was reached for each pig. Dyssynchrony was considered if the TTP delay was above the PEEP 0 cmH_2_O 95^th^ percentile value.

Statistical analysis was performed with JMP version 10 (SAS Institute Inc., North Carolina). Continuous variables are expressed as mean +/− standard deviation (SD) or median with IQR. Repeated measure analysis at various PEEP levels was done with the one-way ANOVA. If a significant difference was found, post-hoc comparisons between the individual PEEP levels was done using Tukey HSD test with Bonferroni correction for p-value. All probability values are 2-sided and a value of ≤0.05 was considered significant, except for multiple comparisons between the different PEEP levels where, according to Bonferroni correction, p < 0.002 was considered significant.

## Results

**Figure 5:**
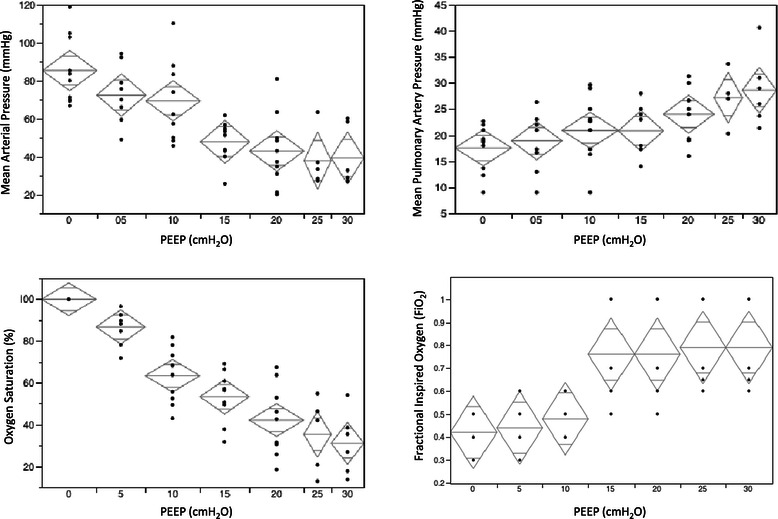
**Change in physiological parameters with escalating PEEP levels.** Mean (+/−95% confidence limits). Mean arterial pressure, mean pulmonary artery pressure, oxygen saturation and fractional inspired oxygen vs PEEP.

Physiological data are presented in Table 1. In summary: with escalating levels of PEEP there was a trend of a fall in MAP and a rise in mean pulmonary artery pressure (MPAP), however only with MAP were significant differences seen between individual PEEP levels: at 15 cmH_2_O and higher vs baseline PEEP 0 cmH_2_O (see Figure 5). Significant hypotension, defined as a decrease in MAP of 20 mmHg, occurred at approximately 15 cmH_2_O PEEP. Higher inspired oxygen levels were required with higher levels of PEEP to maintain oxygen saturation. The step-wise PEEP maneuver was terminated before a PEEP level of 30 cmH_2_0 in four pigs due to life-threatening hypotension and bradycardia.

**Table 1:** **Physiological data (values expressed as mean ± SD)**

PEEP (cmH_2_O)	PEEP 0	PEEP 5	PEEP 10	PEEP 15	PEEP 20	PEEP 25	PEEP 30
Heart rate (bpm)	99 ± 13	104 ± 18	113 ± 26	113 ± 27	109 ± 30	83 ± 39	112 ± 36
Saturation (%)	96 ± 4	97 ± 4	91 ± 7	78 ± 16	73 ± 29	90 ± 15	74 ± 40
Fraction inspired oxygen (FiO_2_)	0.4 ± 0.1	0.4 ± 0.1	0.5 ± 0.1	0.8 ± 0.2	0.8 ± 0.2	0.8 ± 0.2	0.8 ± 0.2
Mean Arterial Pressure (mmHg)	86 ± 18	73 ± 15	70 ± 21	48 ± 11*	43 ± 19*	38 ± 15*	39 ± 15*
Mean Pulmonary Artery Pressure (mmHg)	18 ± 4	19 ± 5	21 ± 6	21 ± 5	24 ± 5	27 ± 5	30 ± 7

*indicates significant difference (p < 0.002) compared with PEEP 0 cmH_2_O.

**Figure 6:**
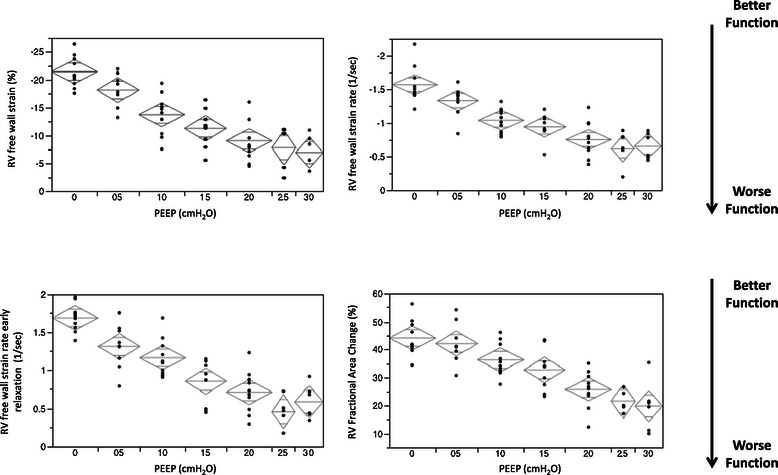
**Change in speckle tracking echocardiography (STE) parameters with escalating PEEP levels.** Mean (+/−95% confidence limits). Right ventricle free wall strain (RVfwS) and strain rate (RVfwSR) are measures of RV systolic function. RVfwS and RVfwSR are negative values indicating tissue contraction: the more negative the value the better the contraction. RV free wall strain rate early relaxation (RVfwSRe) is measure of RV diastolic function and is a positive value: the more positive a value the better the relaxation function.

**Figure 7:**
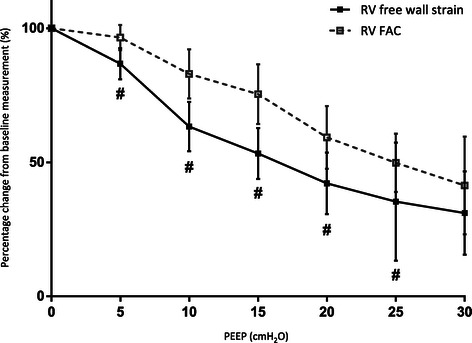
**Percentage change from baseline level in right ventricle free wall strain (RVfwS) and fractional area change (FAC).** RVfwS reduces to a greater extent than FAC and earlier in the PEEP escalating process.

Results of STE analysis (see Figure 6) and conventional echocardiographic analysis of RV function are shown in Table 2. RV systolic function assessed by FAC as well as RVfwS and RVfwSR showed a clear trend towards deterioration with escalating levels of PEEP. RV diastolic function assessed by RVfwSRe also showed a deteriorating trend with escalating PEEP levels. Comparing RVfwS, RVfwSR and RVfwSRe values at different PEEP levels showed a significant difference (p < 0.002 as per the Bonferroni correction) at PEEP 0 cmH_2_O vs PEEP 10 cmH_2_O and above. In addition RVfwS showed a difference at PEEP 5 cmH_2_O vs 15 cmH_2_O and above, RVfwSR and RVfwSRe at PEEP 5 cmH_2_O vs 20 cmH_2_O and above. RVfwSRe also showed a difference at PEEP 10 cmH_2_O vs 25 cmH_2_O and above. FAC only showed a significant difference at PEEP 0 cmH_2_O vs PEEP 20 cmH_2_O and above as well as PEEP 5 cmH_2_O vs 20 cmH_2_O and above. RVfwS decreased to greater extent compared to baseline level, earlier in the step-wise PEEP escalation process (see Figure 7) and significantly decreased before there was a clinical relevant decrease in MAP.

**Table 2:** **Conventional echocardiography and speckle tracking echocardiography data (values expressed as mean ± SD)**

PEEP (cmH_2_O)	PEEP 0	PEEP 5	PEEP 10	PEEP 15	PEEP 20	PEEP 25	PEEP 30
RV EDA (cm^2^)	11 ± 3	11 ± 2	11 ± 1	10 ± 2	10 ± 1	13 ± 2	13 ± 1
RV FAC (%)	44 ± 6	42 ± 7	36 ± 6	32 ± 7	26 ± 7*^⌘^	22 ± 4*^⌘^	20 ± 9*^⌘^
RVfwS (%)	−21.5 ± 3	−18.2 ± 3	−13.7 ± 4*	−11.3 ± 3*^⌘^	−9.0 ± 4*^⌘^	−7.8 ± 4*^⌘^	−6.9 ± 3*^⌘^
RVfwSR (^−1^)	−1.6 ± 0.3	−1.3 ± 0.2	−1.0 ± 0.2*	−0.9 ± 0.2*	−0.8 ± 0.3*^⌘^	−0.6 ± 0.3*^⌘^	−0.7 ± 0.2*^⌘^
RVfwSRe (^−1^)	1.7 ± 0.2	1.3 ± 0.3	1.2 ± 0.3*	0.9 ± 0.3*	0.7 ± 0.3*^⌘^	0.5 ± 0.2*^⌘§^	0.6 ± 0.2*^⌘§^

RV: right ventricle; EDA: end-diastolic area; FAC: fractional area change; RVfwS: right ventricle free wall longitudinal strain; RVfwSR: right ventricle free wall longitudinal free wall strain rate; RVfwSRe: right ventricle free wall longitudinal early strain rate relaxation. NB: RVfwS and RVfwSR are systolic function parameters and are expressed as negative values: the less negative a value, the better the function. RVfwSRe is a diastolic function parameter and expressed as a positive value: the more positive, the better the relaxation function. *indicates significant difference (p < 0.002) compared with PEEP 0 cmH_2_O. ^**⌘**^indicates significant difference compared with PEEP 5 cmH_2_O. ^**§**^indicates significant difference compared with PEEP 10 cmH_2_O.

Analysis of the individual RV free wall segments showed a similar trend of deterioration across segments with escalating PEEP levels (see Table 3). The apical segment did show a significant deterioration at PEEP 10 cmH_2_O and above, whereas the basal and mid segments showed a significant deterioration from PEEP 15 cmH_2_O upwards. Comparing TTP delay values at PEEP 0 cmH_2_O vs final PEEP values, 30% of pigs had dyssynchrony of the RV free wall at the highest PEEP level reached, defined as a delay of >108 msec (as determined by mean TTP at PEEP 0 cmH_2_O + 95% percentile value) between the earliest contracting segment vs the latest contracting segment.

**Table 3:** **Right ventricle free wall strain segmental data (values are expressed as mean ± SD)**

PEEP (cmH2O)	PEEP 0	PEEP 5	PEEP 10	PEEP 15	PEEP 20	PEEP 25	PEEP 30	Mean difference from PEEP 0 to final PEEP
Basal segment S(%)	−19.2 ± 4	−15.8 ± 3	−12.3 ± 5	−8 ± 5*	−9 ± 1*	−7 ± 4*	−6 ± 4*	14.0 ± 1
Mid segment S(%)	−23.4 ± 4	−19.7 ± 5	−15.1 ± 5	−11.2 ± 4*	−8.6 ± 5*^⌘^	−9.3 ± 6*	−8.7 ± 5*^⌘^	15.5 ± 2
Apical segment S(%)	−22 ± 4	−19 ± 4	−12.7 ± 5*	−13.3 ± 3*	−9.3 ± 3*^⌘^	−7.3 ± 3*^⌘^	−7 ± 3*^⌘^	15.2 ± 1
TTP delay (msec)	50 ± 29	78 ± 58	72 ± 28	69 ± 43	113 ± 61	118 ± 133	86 ± 70	71.3 ± 31

S: strain; TTP delay: time to peak strain delay between segmental values. *indicates significant difference (p < 0.002) compared with PEEP 0 cmH_2_O. ^**⌘**^indicates significant difference compared with PEEP 5 cmH_2_O.

### Measurement variability

Blinded interrater variability for STE analysis was assessed by S.G. on a randomly selected pig at all PEEP levels. Bland-Altman analysis demonstrated good intraobserver and interobserver agreement. The interobserver and intraobserver mean difference (± standard error) were respectively: RVfwS −1 (±0.5) and −0.6 (±0.6); RVfwSR −0.1 (±0.1) and −0.1 (±0.1); RVfwSRe 0.1 (±0.1) and 0.1 (±0.1).

## Discussion

Our study demonstrates a clear trend of reduction in RV function with escalating PEEP levels assessed with a conventional echocardiography method, FAC, and with Speckle Tracking Echocardiography (STE), a novel echocardiography technique. Both RV systolic function parameters measured by STE (RVfwS and RVfwSR) and the diastolic function parameter (RVfwSRe) reduced with elevated PEEP levels. A drop of 20 mmHg in the MAP was considered a clinically relevant end-point and this occurred at approximately PEEP 15cmH_2_O. FAC only showed a significant deterioration at PEEP level of 20 cmH_2_O. However STE values of systolic function (RVfwS and RVfwSR) and diastolic function (RVfwSRe) showed significant deterioration earlier in the PEEP escalation process: at PEEP 10 cmH_2_O. No significant difference was seen between either systolic or diastolic parameters at higher PEEP levels suggesting a plateau effect in the degree of RV functional deterioration. Our findings suggest that RV dysfunction induced by PEEP may be identified earlier and with increased sensitivity with STE than by FAC. The effect of PEEP on RV strain has been demonstrated in a study in critically ill patients undergoing a recruitment maneuver [19] indicating the feasibility of this technique in the ICU population.

Echocardiography is an important means of recognizing RV dysfunction induced by mechanical ventilation [6]. Sonographic imaging of the RV can be challenging due to its shape and position and conventional echocardiographic assessment methods are limited by angle-dependence, translational error and often a qualitative approach to analysis. STE is a relatively novel, angle-independent ultrasound imaging technique, which follow groups of grey-scale pixels which create the image of the myocardium (known as ‘kernels’) and tracks their degree of deformation (strain) and rate of deformation (strain rate) as a surrogate for systolic function [7]. Strain is the most commonly utilized STE value clinically, however animal studies have suggested that strain rate may be a more robust measure of myocardial contractility that is less influence by changes in cardiac load and structure and strain may be influenced in particular by afterload [20]. In our study both RVfwS and RVfwSR were both influenced by PEEP to a similar extent. The initial rate of kernels returning to their end-diastolic position (strain rate early relaxation or RVfwSRe) is a surrogate for diastolic function in much the same way as the e’ value with Tissue Doppler Imaging. Although this has not been validated as a clinical reference value at this stage, a small number of animal and clinical studies have shown SRe can identifiy ischaemic areas and viable myocardium in studies of coronary artery disease [21] where diastolic as well as systolic dysfunction occurs.

Unlike the LV, which contracts in all planes (longitudinally, radially, circumferentially with twist and torsion [22]) the RV contracts predominantly in the longitudinal direction due to the dominance of longitudinal muscle fibers in the RV free wall [23]. This places RV free wall strain, which assesses motion in the longitudinal direction, as a sensitive, quantifiable and importantly a feasible tool for assessing RV function non-invasively. Indeed RV free wall strain has been investigated in pulmonary hypertension cohorts and trumps all other echocardiographic methods in predicting both symptom progression and mortality [10,13].

PEEP is an integral part of mechanical ventilation particularly in the critically ill patient with acute lung injury or ARDS. Counteracting alveolar cycling, collapse, derecruitment and to maintain functional residual capacity PEEP aims to reduce hypoxaemia and ventilator-induced lung injury [16]. High levels of PEEP are often recommended in severe ARDS [24] and can affect biventricular function in a variety of complex methods. The exact physiological effects of PEEP on haemodynamics are not entirely elucidated, however RV dysfunction and reduced cardiac output are of serious concern, with cor pulmonale reported in 20-25% of patients with ARDS [2,25] and is associated with significantly higher mortality [1]. The effect of PEEP on the right ventricle depends on the changes in lung volumes and intrathoracic pressure as well as the underlying pathological state and the physiological response of the pulmonary vasculature [17]. PEEP is reported to predominantly affect RV afterload resulting in a reduced RV stroke volume through increased RV outflow impedance in ARDS patients [26,27] and there are reports of increased RV end-diastolic area [25]. This has led to the concept of a ‘RV protection’ approach to mechanical ventilation in ARDS patients, which limits PEEP and avoids hypercapnic acidosis [28]. STE potentially may provide a method for identifying RV failure induced by PEEP ahead of conventional methods of RV function assessment. This has the potential to allow the physician to direct therapy earlier at protecting the RV [25].

### Limitations

STE, as with conventional echocardiography, is limited by adequate image quality. We utilized ICE in order to maximize the imaging quality of the RV free wall as neither transthoracic or transoesophageal echocardiography could reliably be performed to provide sufficient image quality of the RV free wall for STE analysis. This relates to the mediastinal anatomy of the pig model. The use of STE with ICE has not been validated, however STE analysis is angle-independent, was feasible and each pig acted as its own control. The ultrasound equipment is comparable and the only difference is the transducer. Our data should be translatable to echocardiography images acquire by other transducers. However, the need to use ICE prevented many of the standard echocardiography measures of RV function such as TAPSE and Sm by Tissue Doppler Imaging as these values are angle dependent and require apical imaging. FAC was the most plausible method to assess RV function as recommended by ASE guidelines [29]. Tachycardia can also impair the software’s ability to accurately track the speckles of the image, and heart rates greater than 100 were frequently observed particularly during the escalating PEEP process. We performed the step-wise PEEP escalation process in pigs with healthy lungs, pigs with diseased lung and reduced compliance may affect results.

## Conclusion

RV dysfunction in the critically ill is known to be associated with poor outcomes and can be induced by mechanical ventilation and PEEP therapy. Speckle tracking echocardiography is a quantifiable, sensitive and feasible angle-independent method for detecting RV dysfunction induced by escalating PEEP levels, and may display dysfunction ahead of conventional echocardiographic methods of assessment. The STE software is available on most current high-end machines, and is becoming increasingly available in intensive care units world-wide. Further studies in the ICU population, particularly with acute lung injury and ARDS, using transthoracic imaging are warranted.

## Abbreviations

ARDS: Acute respiratory distress syndrome
EDA: End-diastolic area
FAC: Fractional area change
FiO2: Fraction of inspired oxygen
ICE: Intracardiac echocardiography
IQR: Interquartile range
MAP: Mean arterial pressure
MPAP: Mean pulmonary artery pressure
PEEP: Positive end expiratory pressure
RV: Right ventricle
RVfwS: Right ventricle free wall longitudinal strain
RVfwSR: Right ventricle free wall longitudinal free wall strain rate
RVfwSRe: Right ventricle free wall longitudinal early strain rate relaxation
S: Strain
SD: Standard deviation
STE: Speckle tracking echocardiography
TTP delay: Time to peak strain delay between segmental values
